# Detection of *Clostridium sporogenes* in a Roman-era cattle mass grave at Vilauba

**DOI:** 10.1080/21505594.2025.2580731

**Published:** 2025-10-27

**Authors:** Daniel Anton Myburgh, Nicolas Antonio da Silva, Magdalena Haller-Caskie, Lídia Colominas, Pere Castanyer, Joan Frigola, Joaquim Tremoleda, Christina Hölzel, Daniel Unterweger, Almut Nebel, Ben Krause-Kyora

**Affiliations:** a Institute of Clinical Molecular Biology, Kiel University, Kiel, Germany; b Catalan Institute of Classical Archaeology, Tarragona, Spain; c Grup de Recerca Arqueològica del Pla de l’Estany, Banyoles, Spain; d Museu Arqueològic de Banyoles, Banyoles, Spain; e Institute for Animal Breeding and Husbandry, Faculty of Agricultural and Nutritional Sciences, Kiel University, Kiel, Germany; f Institute of Experimental Medicine, Kiel University, Kiel, Germany; g Max Planck Institute for Evolutionary Biology, Plön, Germany

**Keywords:** Ancient DNA, bacterial infections, bovine, clostridial myonecrosis, *Clostridiaceae*, *Mycobacteriaceae*, phylogenetic analysis

## Abstract

In the ancient Roman world, cattle played an integral role in daily agricultural tasks, providing the means necessary to plow fields, mill grains, and transport goods. The research presented here deals with the remains of 14 cattle discovered in a mass grave at the Roman villa of Vilauba in Catalonia, Spain. According to the archaeological record, it can be ruled out that the animals were slaughtered for consumption, banqueting, or sacrificial purposes. By investigating the metagenomic sequences generated from the bovine remains, we identified in three individuals a group I *Clostridium* strain, phylogenetically related to known producers of botulinum neurotoxins – suggesting that the Vilauba strain may have had toxigenic potential. Moreover, we discovered a *Mycolicibacterium* species phylogenetically related to known opportunistic pathogens. While no definitive conclusions can be drawn about disease, the phylogenetic placement of these taxa and the detection of *Clostridium* virulence-associated genes suggest a possible role beyond postmortem contamination. Collectively, these findings draw attention to atypical bacterial species, such as *C. sporogenes*, which are often overlooked in palaeogenomic studies due to their ambiguous status as environmental microbes, commensals, or potential pathogens. Their detection in animal remains highlights that they may represent a blind spot in our current understanding of livestock health. More broadly, this study underscores the current complexity of investigating such taxa and emphasizes the need for novel methods to disentangle the roles of these bacterial species.

## Introduction

The archaeological site of the Roman villa of Vilauba is situated in the northeastern corner of the Iberian Peninsula in Catalonia, Spain. Archaeological evidence indicates that Vilauba was inhabited from the 1st century before the Common Era (BCE) to the 7th century CE [1]. The arable farmland surrounding the villa is estimated to have been between 50 to 85 hectares and agricultural activities included the growing of grains, legumes, olives, and grapes [1,2]. Breeding of livestock was practised as well and the inhabitants made use of these domesticated animals for traction and various products.

Recent excavation of a pit at Vilauba unearthed 14 disarticulated cattle skeletons [2]. The particular characteristics of this assemblage, made in a very short period of time and following completely different patterns to those we know in other similar archaeological and chronological contexts, allow us to consider different hypotheses about the causes that motivated the formation of this mass grave. Osteological examination revealed they were adults of different ages, from 2–3 to 8–10 y old [2]. Further investigation of the remains showed that the assemblage consisted of carcasses from bulls, cows, and at least one ox. Considering their varied ages and sexes, these cattle are thought to be representative of a typical herd. Cut marks found on 12.3% of the skeletal elements suggest that the carcasses were skinned and defleshed, prior to being dumped into the pit [2]. However, the manner in which the cattle were processed appears to differ from the standard Roman butchery practices [2–5]. The bones were not fractured, which differs greatly from the usual Roman processing practices. At the same time, it also differs from the practices documented in other assemblages recovered from the same Roman villa of Vilauba. Therefore, there is no archaeological evidence that the cattle were killed for routine consumption or as part of a ceremonial feast or religious sacrifice [2]. Thus, the abnormal circumstances surrounding the death of these 14 cattle are highly suggestive of a disease outbreak among the herd. Notably, clinical signs attributed to several cattle diseases have been indirectly described in the writings of prominent Roman scholars (Columella, De re rustica, VI, 5–15; Varro, De re rustica, II, 5; Pliny the Elder, Naturalis Historia, VIII; Palladius, Opus Agriculturae, I and XIV).

In this study, we used a palaeogenomic approach to investigate whether the Vilauba cattle had contracted an infectious disease that could explain their deaths. To this end, we performed an untargeted pathogen screening on the remains of all 14 animals. Our findings revealed the presence of a strain of *Clostridium* and one or more strains of *Mycolicibacterium*. Our results extend current understanding of these bacteria and their possible roles as disease-causing agents in cattle and provide first-order information on the management of livestock disease in Roman times.

## Materials and methods

### Archaeological context and analyzed samples

Excavation of a pit at the Roman villa of Vilauba (Catalonia, Spain) unearthed the disarticulated skeletons of 14 cattle [2]. Osteological age-at-death analysis of the cattle revealed that one was 2–3 y old, two were 8–10 y old, and the remainder were 6–8 y old [2]. Additionally, it was shown that the skeletal material belonged to both bulls and cows. In this study, 14 left tibiae and 14 molars from 14 left mandibles were analyzed (Supplementary Table S1). As the skeletons of these 14 animals were found disarticulated in the pit, it was impossible to match the tibiae to their corresponding molars and to identify the individuals to which the bones belonged.

### Sample preparation and DNA extraction

To minimize contamination, all laboratory work was carried out within the dedicated clean rooms of the Ancient DNA Laboratory at Kiel University, following strict guidelines [6,7]. All surfaces were thoroughly cleaned with sodium hypochlorite or nucleic acid removal reagents and sterilized using UV radiation before and after use. Laboratory personnel wore disposable coveralls, gloves, and facemasks at all times. All reagents were DNase, RNase, and pyrogen free and only filtered pipette tips were used. Following a previously published protocol [8], samples were washed in pure sodium hypochlorite and then rinsed with distilled water. Afterward, they were dried overnight in an incubator at 37°C and powdered using an electric drill (for tibiae samples) or a ball mill homogenizer (for molar samples). Between 80–120 mg of powder was then incubated overnight with 960 µl 0.45 M, pH 8 ethylenediaminetetraacetic acid, and 40 µl 0.25 mg/ml proteinase K at 37°C with mild rotation. In addition, negative extraction controls were included. Subsequently, DNA extraction was carried out using a published silica adsorption protocol [9].

### Library preparation and sequencing

Double-stranded, half-UDG-treated libraries were generated following previously published work [8,10]. Negative library controls were also included. Libraries were then shotgun sequenced at the Institute of Clinical Molecular Biology at Kiel University, using the Illumina HiSeq 6000 (2x100 bp) platform. All samples were shotgun sequenced in one-hundredth of a lane. This initial sequencing generated about 15 million reads per sample. During preliminary screening, if we detected many sequences mapping to either the cattle or *Clostridium* references, we opted to generate more reads. Hence, four samples had more reads generated by “deeper” shotgun sequencing. Sample KB210573 on half of a lane, KB210591 and KB210592 each on a tenth of a lane and KB210593 on a quarter of a lane and a tenth of a lane. The samples are referred to as “deeper sequenced” and are denoted with a “*” symbol in Supplementary Table S1. Subsequently, raw sequence reads were processed using Clip&Merge v1.7.8 [11] with default settings.

### Genetic sex determination

To determine genetic sex, reads generated from the cattle samples were mapped to the bovine reference genome BosTau9 (National Center for Biotechnology Information (NCBI) Genome Assembly: ARS-UCD1.2). Since BosTau9 is derived from a female specimen, we supplemented the Y chromosome sequence from BosTau7 (NCBI Genome Assembly: Btau_4.6.1) to the reference FASTA file used for mapping, following the method applied in previous studies [12,13]. Reads generated from the Vilauba cattle samples were then mapped to this reference using the Burrows-Wheeler Aligner (BWA) aln algorithm [14]. Only reads with a mapping quality score greater than 30 were included in the analysis. Only samples that met the threshold of 15,000 reads after quality filtering were chosen for analysis. To infer genetic sex, we applied the method outlined by Mittnik et al., (2016) [15], which relies on calculating the ratio of reads mapped to the X chromosome compared to autosomal coverage (Rx). Since this method was originally developed for ancient human samples, we adapted the script to accommodate the number of chromosomes in the bovine genome.

### Bovine mitochondrial haplogroup analysis

To determine the mitochondrial DNA (mtDNA) haplogroup, we aligned the reads generated from the samples to a *Bos taurus* mitochondrial reference genome (NCBI GenBank: V00654.1) with BWA aln [14]. Variant calling was performed using BCFtools v1.12 [16] with a base quality and mapping quality threshold of 30 and the ploidy set to 1. Uncovered positions were masked with “N” during the consensus sequence generation. The consensus sequence from sample KB210573 was concatenated with sequences from 158 publicly available bovine mtDNA samples [17], representing the diversity of modern cattle lineages (Supplementary Table S4). All sequences were aligned with Multiple Alignment using Fast Fourier Transform (MAFFT) v7.505 [18]. A phylogenetic tree was constructed with Randomized Axelerated Maximum Likelihood (RAxML) v8.2.12 [19] using the following parameters: -f a -x 12345 -p 12345 -# 100 -m GTRGAMMA. Tree files were annotated via the online Interactive Tree of Life (iTOL) v6 [20].

### Metagenomic pathogen screening, mapping, and quality filtering

Using the Megan Alignment Tool (MALT) v0.4.1 [21] in BlastN mode with semi-global alignment and a minimum percent identity of 95%, processed reads were aligned against a curated reference database comprising 27,730 complete bacterial and 10,543 viral genomes downloaded from NCBI [22]. The MALT alignment was then visualized with the Metagenome Analyzer 6 (MEGAN6) v6.25.9 [23].

Samples positive for bacterial or viral reads were mapped to relevant references with BWA aln [14]. Duplicate reads were removed with DeDup v0.12.9 [11]. After mapping, DamageProfiler v.1.1 [24] was used to construct DNA deamination plots and SAMtools v1.10 [25] was utilized to generate mapping statistics. Read alignments were visualized using Integrative Genomics Viewer (IGV) v2.17.1 [26] and reads were entered on the Basic Local Alignment Search Tool (BLAST) v2.15.0 [27] to assess their identity to the reference.

After mapping to mycobacteria references, quality filtering was conducted to remove low-quality reads. SAMtools v1.10 [25] was utilized to remove reads with a minimum quality below 30 and a percent identity filter of 95% was applied using filterSAM v0.0.10 [28].

### Phylogenetic analysis

Modern bacterial genomes used for constructing the phylogenetic trees were downloaded from the NCBI database (Suplementary Tables S12 and S13). Thereafter, these genomes were converted from FASTA to FASTQ format with the wgsim tool from the SAMtools v1.10 [25] software package. For this conversion, the rate of mutations, fraction of indels, the probability that an indel is extended and base error rate were all set to zero. All FASTQ files were subsequently mapped against the respective type-strain references either *Clostridium botulinum* ATCC 3502 (National Center for Biotechnology Information Reference Sequence (NCBI RefSeq): NC_009495.1) or *Mycobacterium tuberculosis* H37Rv (NCBI RefSeq: NC_000962.3) with BWA aln [14]. BAM files generated from the mapping were then converted to VCF format, with SAMtools v1.10 [25], Picard tools v2.9.0–24 [29] and the Genome Analysis Toolkit v4.4.0.0 [30]. Next, MultiVCFAnalyzer v0.85.1 [31] was utilized to generate SNP-based alignments, with the following parameters: true for writing allele frequencies, minimal genotyping quality of 30, minimal coverage for base call of 3 and 0.9 minimal allele frequencies for homozygous and heterozygous calls. Thereafter, a phylogenetic tree was generated using RAxML v8.2.12 [19] with the following parameters: -f a -x 12345 -p 12345 -# 1000 -N 1000 -m GTRGAMMA. Tree files were annotated via the online Interactive Tree of Life (iTOL) v6 [20].

### Virulence factor analysis

To carry out an analysis of virulence factors, the relevant gene sequences were downloaded from the NCBI [22] or Virulence Factor Database (VFDB) [32] database. *Clostridium*-specific virulence genes (belonging to *Clostridium* species other than *C. sporogenes*) are listed in VFDB, the corresponding genes were additionally retrieved from various annotated *Clostridium* genomes available in NCBI. Thereafter, the generated cattle sequences were mapped against these genes with BWA aln [14]. IGV v2.17.1 [26] was employed to manually view the alignment of reads to genes. BLAST v2.15.0 [27] was used to check the identity of the reads to the *Clostridium* species. The alignment of the reads to specific genes was viewed with NCBI Nucleotide Graphics. Lastly, the percentage coverage of the virulence genes was plotted in a heatmap using R v4.3.1 [33] packages ggplot2 v3.4.3 [34] and reshape2 v1.4.4 [35]. Only virulence genes which were covered in at least one sample and only samples which showed coverage of at least one virulence gene were included in the heatmap. Only the highest percentage coverage is displayed for each of the tested virulence genes from the different *Clostridium* species.

### Ethics statement

This study was conducted in full compliance with the ethical principles of DNA research on historical remains, as outlined by Alpaslan-Roodenberg et al., (2021) [36]. Permission to collect and analyze the skeletal remains used in this research was granted by the respective curators, archaeologists, and their affiliated institutions. Archaeological samples were acquired directly from these representatives. Authors Lídia Colominas, Pere Castanyer, Joan Frigola, and Joaquim Tremoleda, affiliated with the Catalan Institute of Classical Archaeology, Grup de Recerca Arqueològica del Pla de l’Estany, and Museu Arqueològic de Banyoles, respectively, were directly involved in obtaining permissions and analyzing the remains. All other modern genomic data used in this study were obtained from either the National Center for Biotechnology Information or the Virulence Factor Database, both of which are publicly available and allow unrestricted reuse under open licenses.

## Results

### Cattle genetic sex and mtDNA analysis

**Figure 1.**
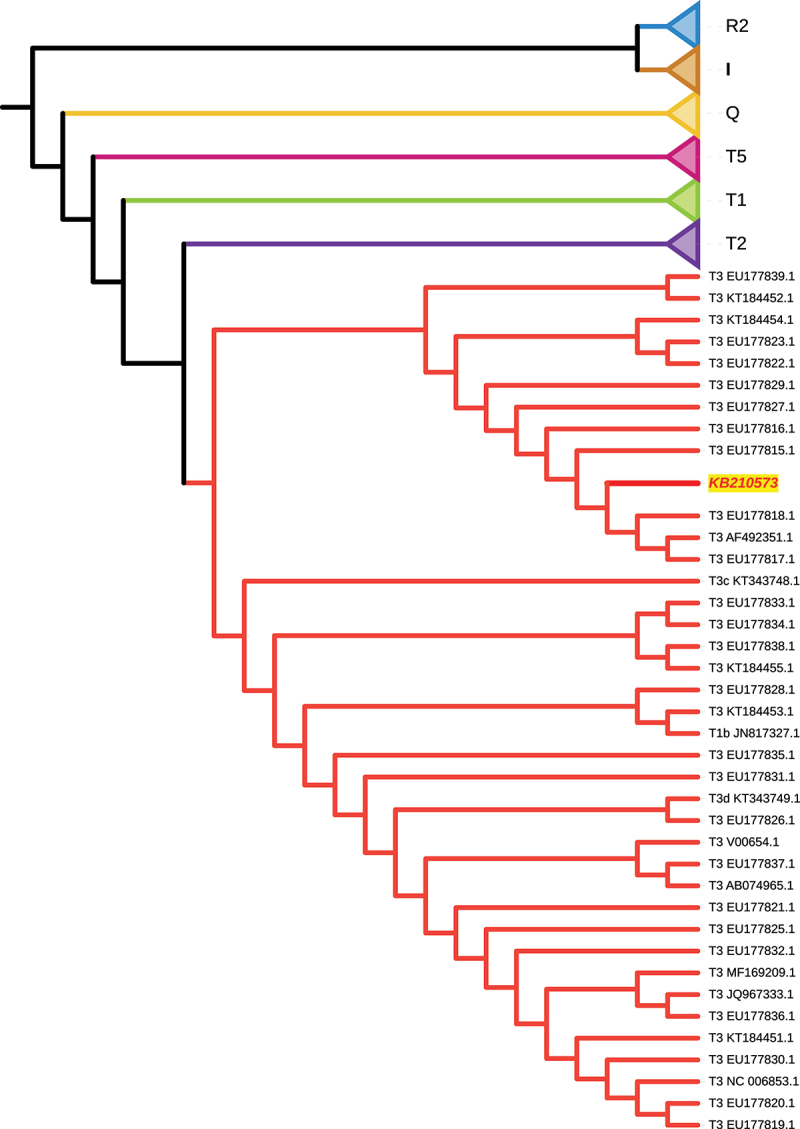
Phylogenetic analysis of bovine mitochondrial haplogroups. Maximum likelihood phylogenetic tree based on mitochondrial DNA (mtDNA) sequences, depicting the position of our ancient sample (KB210573 highlighted in yellow) within the broader mtDNA diversity of modern cattle populations. Leaf labels describe the assigned mtDNA haplogroups of modern samples and their corresponding sequence accessions (Supplementary Table S4). Branch lengths are not scaled for simplicity, and all clades except T3 are collapsed. A more detailed version, including branch lengths and expanded clades, is available in Supplementary Figure S2.

For this investigation, a left tibia and a left lower molar from each of the 14 cattle excavated at the Roman villa of Vilauba were sampled (Supplementary Table S1). The carcasses of these 14 animals were recovered disarticulated in a pit, making it impossible to match a specific tibia to a corresponding molar and to determine to which individual the bones belonged. One molar sample (KB210586) had no measurable DNA concentration and was not selected for further analyses (Supplementary Table S1), thus a total of 27 samples were used for this study. After shotgun sequencing, the sequences generated from 10 samples had sufficient reads to meet the threshold for determining genetic sex (Supplementary Table S1, Supplementary Table S2). The genetic sex results for these 10 samples confirmed prior osteological findings and indicated that the skeletal assemblage comprised material from both bulls and cows. Moreover, previously published osteometric data identified samples KB210576 and KB210578 as male [2], which was corroborated by the genetic sex (Supplementary Table S1, Supplementary Table S2). Analysis of mtDNA revealed that sample KB210573 had sufficient coverage to generate a reliable consensus sequence (meeting the criteria of >99% breadth of coverage and an average depth >10x) that could be assigned to sub-haplogroup T3 (Figure 1, Supplementary Table S2, Supplementary Table S3). However, due to poor mtDNA preservation in the remaining samples, no other mitochondrial haplogroups could be determined. Kinship or population genetic analyses that require genome-wide data were also not possible.

### Pathogen screening

Metagenomic pathogen screening was conducted using sequences generated from all 27 bovine tibiae and molar samples. Screening highlighted one *Clostridium* species in a single sample and three mycobacteria species which had reads assigned to them across multiple samples (Supplementary Table S5). Conversely, screening for bovine-specific viral pathogens did not reveal the presence of any detectable virus sequences. Subsequently, we conducted mappings of the bovine sequences against references of the *Clostridium* and mycobacteria species detected during screening. Results indicated that all four of these references displayed deamination patterns associated with ancient DNA (aDNA) (Supplementary Figures S3a-d, mapping statistics shown in Supplementary Table S6). Mappings conducted against well-characterized bovine viral pathogens (such as bovine viral diarrhea virus, foot, and mouth disease virus, and bluetongue virus) revealed no positive results.

### *Clostridium* identification and phylogenetics

To further investigate the *Clostridium* reads detected in the cattle samples, all sequences were mapped against 73 *Clostridium* references, as well as 5 species which were formerly classified within the *Clostridium* genus (Supplementary Table S7). Mapping results showed that *Clostridium sporogenes* AM1195 (NCBI RefSeq: NZ_CP013701.1) was the best-covered reference in the two samples KB210573 and KB210593 (Supplementary Table S8). Moreover, the reads aligned to the reference displayed deamination patterns characteristic of aDNA (Supplementary Table S3a). Since KB210573 was taken from a bull tibia and KB210593 from a bull molar, they could theoretically come from the same animal. In addition, *Clostridium* reads were also present in sequences generated from three other samples (Suplementary Table S1). Thus, the minimum number of individuals (MNI) with *Clostridium* reads is three.

**Figure 2.**
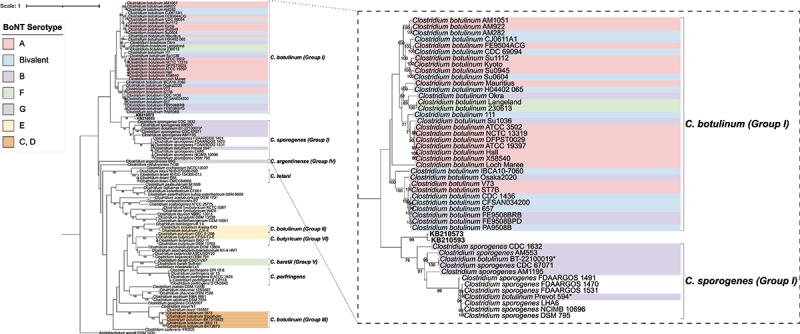
Phylogenetic analysis of *Clostridium* species. Maximum likelihood tree based on whole genome single nucleotide polymorphisms (SNPs). SNPs were derived after mapping all genomes against *Clostridium botulinum* ATCC 3502 (NCBI RefSeq: NC_009495.1). The tree is rooted using the outgroup *Acetobacterium woodii* DSM 1030 (NCBI RefSeq: NC_016894.1). The scale bar represents the number of nucleotide substitutions per site. Bootstrap values greater than 70% are displayed numerically on branch nodes. Cattle samples KB210573 and KB210593 are marked in bold. Legend colors indicate the corresponding botulinum toxin serotype. *, denotes that our findings suggest that *Clostridium botulinum* BT-22100019 (NCBI RefSeq: NZ_CP121696.1) and *Clostridium botulinum* Prevot 594 (NCBI RefSeq: NZ_CP006902.1) may have been previously misassigned as *C. botulinum* instead of *C. sporogenes*. Genomes used for constructing the phylogenetic tree are listed in Supplementary Table S12.

Next, we generated a maximum likelihood tree using the two best-covered cattle samples (KB210573 and KB210593) and representative modern *Clostridium* genomes. The bovine samples were positioned between the *Clostridium botulinum* group I and the *Clostridium sporogenes* clades (Figure 2). Upon closer inspection of the *C. sporogenes* strains, it became apparent that they were split into two clades, with one clade consisting of strains which can produce botulinum neurotoxins (BoNTs), whilst the other comprised predominantly nontoxic strains (Figure 2).

### Analysis of *Clostridium* virulence genes

To examine pathogenicity, we mapped the reads generated from all bovine samples against 364 clostridial virulence factors from the NCBI database [22] (Supplementary Table S9) and all *Clostridium*-specific virulence factors from the VFDB database [32]. Since VFDB provides a list of *Clostridium*-specific virulence genes, these genes were also retrieved from annotated *Clostridium* genomes available on NCBI (thus constituting the 364 virulence genes shown in Supplementary Table S9). The outcome of these mappings revealed that reads generated from sample KB210573 displayed coverage of the clostridial virulence genes linked to cell adhesion and invasion, such as chaperonin GroEL [37–39] and the NFACT RNA binding domain which encodes for fibronectin-binding protein A, an adhesin [40–44] (Supplementary Table S4). Additionally, there was coverage of several virulence genes involved in the degradation of host extracellular matrix components, including clostripain which encodes a cysteine protease [45–47], collagenase [48–50] and zinc-dependent phospholipase C family protein which is implicated in host cell rupture [51,52] (Supplementary Table S4). Coverage of genes encoding hemolysins which are involved in erythrocyte destruction was also noted, such as hemolysin III family protein and hemolysin family protein [53] (Supplementary Table S4). Lastly, we demonstrated coverage of two genes linked to bacterial stress responses. These genes encode the type II toxin-antitoxin system PemK/MazF family toxin [54–58] and zeta toxin family protein [59,60] (Supplementary Table S4). In addition, reads generated from sample KB210591 showed coverage of the gene encoding the chaperonin GroEL gene (Supplementary Table S4).

### *Mycolicibacterium* identification and phylogenetics

The cattle sequences were also mapped against 48 references from the *Mycobacteriaceae* family (Supplementary Table S10). After quality filtering all samples, the highest genome coverage was observed in KB210573 and KB210593 mapped to *“Mycobacterium gallinarum”* JCM 6399 (NCBI RefSeq: NZ_AP022601.1; quotation marks indicate that this name is not yet validly published under the International Code of Nomenclature of Prokaryotes) and *Mycolicibacterium gadium* JCM 12,688 (NCBI RefSeq: NZ_AP022608.1). The aligned reads from these two samples also displayed DNA deamination patterns characteristic of a DNA (Supplementary Table S11, Supplementary Figures S5a, S5b). Moreover, we found reads matching “*Mycobacterium gallinarum”* JCM 6399 and *Mycolicibacterium gadium* JCM 12,688 in all *Clostridium-*positive samples.

**Figure 3.**
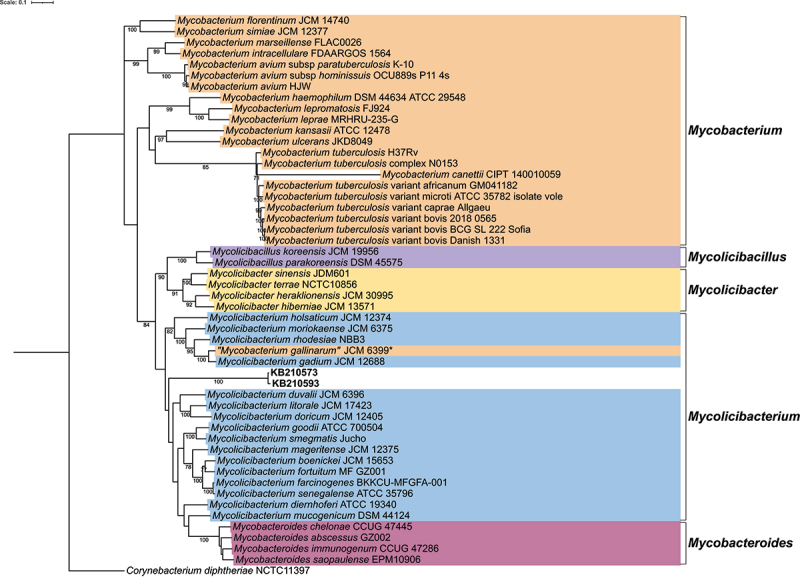
Phylogenetic analysis of mycobacteria species. Maximum likelihood tree based on whole genome single nucleotide polymorphisms (SNPs). SNPs were derived after all genomes and samples were mapped against *Mycobacterium tuberculosis* H37Rv (NCBI RefSeq: NC_000962.3). The tree is rooted using the outgroup *Corynebacterium diphtheriae* NCTC11397 (NCBI RefSeq: NZ_LN831026.1). The scale bar represents the number of nucleotide substitutions per site. Bootstrap values greater than 70% are displayed numerically on branch nodes. Cattle samples KB210573 and KB210593 are marked in bold. Legend colors correspond to the labeled genus. *, denotes that our findings suggest that “*Mycobacterium gallinarum”* JCM 6399 (NCBI RefSeq: NZ_AP022601.1) may have been previously misassigned and is more appropriately classified as *Mycolicibacterium gallinarum* JCM 6399. Genomes used for constructing phylogenetic tree are listed in Supplementary Table S13.

Lastly, we constructed a maximum likelihood phylogeny, using the two best-covered cattle samples (KB210573 and KB210593) and genomes representative of each of the five mycobacteria genera proposed by Gupta et al., (2018) [61] (Figure 3). Samples KB210573 and KB210593 were positioned within the *Mycolicibacterium* clade (Figure 3).

## Discussion

In this study, we investigated a total of 27 molar and tibia samples obtained from the remains of 14 cattle uncovered at the Roman villa of Vilauba in Catalonia, Spain (Supplementary Table 1). Overall, the endogenous DNA preservation of these samples is poor, as demonstrated by the best-preserved sample (KB210573) having a 1x coverage of 4.32% of the reference bovine genome (Suplementary Table S2). During the archaeological excavation of the pit, it was shown that the bones in the upper layer were affected by roots and water as well as by carrion animals [2]. This observation suggests that the cattle carcasses were left uncovered for some time, likely hastening the DNA degradation in the remains. Moreover, as the skeletal material was found disarticulated and commingled, this made it impossible to match the tibiae to their corresponding molars and to determine to which individuals they belonged. Genetic matching of molar and tibiae was also not possible due to the poor preservation. Despite these challenges, the genetic sex was successfully determined for 10 samples and demonstrated that there were skeletal elements belonging to both bulls and cows (Supplementary Table S1, Supplementary Table S1, Supplementary Table S2). Furthermore, one sample (KB210573) could be reliably classified into bovine mitochondrial sub-haplogroup T3 (Figure 1, Supplementary Table S3), which is today the dominant haplogroup among European cattle [62,63]. Interestingly, phylogenetic analysis revealed that the mtDNA of KB210573 belonged to a clade consisting of several Italian breeds and no Spanish breeds (Figure 1, Supplementary Table S2). These Italian breeds, such as the Chianina, Maremmana, and Piedmontese, are prized for their large sizes and have been traditionally bred for draught purposes and beef [64]. It is therefore feasible that some of the cattle at Vilauba were an Italian breed which were brought to Catalonia by the Romans to carry out agricultural labor at the villa [65,66]. Osteological analysis demonstrated that the average wither heights of these 14 cattle uncovered from the pit were slightly higher than those recorded from other cattle remains uncovered at the villa, confirming that they were indeed a larger breed [2]. Enlargement of the medial trochlea was noted in at least two metatarsals and one metacarpal, inferring that these animals were frequently used for draught purposes [2].

Our analyses have uncovered the presence of ancient *Clostridium* and *Mycolicibacterium* DNA in the remains of at least three cattle (Supplementary Table S1, Supplementary Table S8, Supplementary Table S11, Supplementary Table S3a, Supplementary Figures S5a, S5b). Subsequent phylogenetic analysis of the ancient *Clostridium* reads indicated a close relationship between the bovine samples and clades of BoNT-producing *C. sporogenes* strains and *C. botulinum* group I (Figure 2). This observation infers that the *Clostridium* species in the Vilauba cattle was likely either a *C. botulinum* strain belonging to group I or a *C. sporogenes* strain. We additionally observed that the *C. sporogenes* strains form two clades based on toxicity and that our cattle sequences were more closely related to the toxin-forming clade (Figure 2). Phylogenetic investigation of the ancient mycobacteria reads revealed that the cattle samples were positioned in the *Mycolicibacterium* clade, related to several species described as opportunistic pathogens and implicated in sporadic infections [67,68] (including *Mycolicibacterium holsaticum* [69], *Mycolicibacterium doricum* [70], *Mycolicibacterium moriokaense* [71], *Mycolicibacterium littorale* [72], *Mycolicibacterium rhodesiae* [73]) (Figure 3). The *Mycolicibacterium* genus is highly diverse, consisting of both nonpathogenic commensals and rapid-growing atypical pathogens [61,74]. In cattle, some of these pathogenic species have been implicated in granulomatous inflammatory disease of the skin lymphatics [75,76], as well as mastitis [77] and respiratory infections [78]. *Mycobacterium avium* subsp. *paratuberculosis* is related to cattle disease as well, being the causative agent of paratuberculosis (Johne’s Disease), a chronic wasting disease that may end up with watery diarrhea and cachexia in elder animals, with some animals losing weight until death [79]. However, MALT hits for *Mycobacterium avium* subsp. *paratuberculosis* (Supplementary Table S5) were false positives caused by shared sequence homology with *Mycolicibacterium*.

The detection of group I *Clostridium* (likely *C. sporogenes*) DNA in three individuals raises interesting questions about its potential impact on cattle health and its broader association with cattle. This species, while not a well-described cattle pathogen, has been associated with human cases of botulism [80–82], bacteremia [83,84], septicemia [85], septic arthritis [86] and clostridial myonecrosis in humans and horses [87–90]. Additionally, *C. sporogenes* produces thiaminase I, an enzyme which degrades thiamine [91–93]. In turn, thiamine deficiency has been implicated in the development of cerebrocortical necrosis, a deadly neurological condition in ruminants [94–96], though a causal role of bacterial thiaminases in disease etiology is doubted [97]. Among the aforementioned types of disease, botulism is the most frequent one in cattle and outbreaks may affect hundreds of animals [98]. Most bovine cases of botulism are caused by BoNT type D, C, or a mosaic variant [98], but there are also cases related to BoNT type B [99,100], which is a serotype shared between *C. botulinum* and *C. sporogenes* [80]. Although no BoNT genes were detected in our sequencing data – likely due to poor DNA preservation – the Vilauba strain’s phylogenetic placement between known BoNT-producing *C. sporogenes* and *C. botulinum* strains (Figure 2) suggests it may have carried such genes. Interestingly, the Vilauba strain harbored genes linked to cell adhesion, invasion, and the destruction of tissues and erythrocytes (Supplementary Figure 4), which may indicate a capacity for localized infection. A recent study from Siberia implicated *C. sporogenes* in multiple cases of bovine enteritis and enterotoxemia affecting cows, heifers, and calves [101], suggesting that its role as a livestock pathogen may be underrecognized. These collective findings indicate that further investigation is warranted to clarify the potential role of *C. sporogenes* as a causative agent of bovine disease, especially under specific environmental or physiological conditions.

Several species within the *Clostridium* genus are recognized as members of the mammalian gut microbiome [102,103]; however, their specific roles in metabolite production and contributions to host health remain poorly understood. The detection of *Clostridium* DNA in the remains of at least three cattle, dating back approximately 2000 y is highly unexpected, as gut microbiome bacteria are rarely detected in tooth and bone material. The discovery of this bacterial DNA in tibiae and molar samples suggests that bacteria infiltrated these elements either ante- or postmortem. Multiple *Clostridium* species are capable of entering bone or dental tissue via systemic bloodstream infections [104,105]. Alternatively, the DNA detected here may have been released from the gut and introduced to the bones and teeth through leaching during taphonomic processes. However, several lines of evidence argue against this explanation. Archaeological findings, including cut marks on the medial side of two carcases, suggests that at least some animals were eviscerated prior to being dumped in the pit [2], likely reducing the presence of gut-associated bacteria. The assemblage was also disarticulated and commingled – conditions that should have favored widespread microbial transfer – yet *Clostridium* DNA was detected in only three individuals. If gut-derived contamination were the sole source, then we would expect *Clostridium* DNA to appear in high abundance across the majority of our samples and in other archaeologically comparable contexts. However, this was not the case, and we have also not detected *C. sporogenes* sequences in any cattle remains from other archaeological sites (unpublished). Taken together, this restricted detection pattern, when considered alongside the archaeological context of a mass grave, argues against generalized gut-derived taphonomic contamination and is more consistent with an antemortem presence of *C. sporogenes*.

On the other hand, the detection of *C. sporogenes* DNA in these remains may simply reflect postmortem environmental infiltration from soil or water sources, rather than active infection. This bacterium is known to inhabit soil and water and is capable of forming resilient spores that persist under harsh conditions [106,107]. Although several features of our assemblage, such as the limited detection in only a few individuals, the archaeological context of a mass culling, and the absence of *C. sporogenes* in other similarly treated remains argue against widespread contamination, it remains possible that *C. sporogenes* entered select carcasses opportunistically after burial. Such infiltration would have been more likely in cases where soft tissues or bone structures were damaged, compromising their integrity. Moreover, given its spore-forming capacity, dormant spores in the soil may have proliferated under the favorable conditions within the mass grave. Overall, this scenario underscores a broader challenge in current aDNA research, which is the inability to clearly distinguish between environmental contaminants, commensals, and genuine pathogens. As a result of this challenge, many studies choose to focus on pathogens with clearer clinical profiles, such as those known to cause primary infections or that are rarely found in soil or the gut. While this is a practical and conservative approach, it may also contribute to a critical underrepresentation of the true diversity of infectious agents in ancient populations. Future research should therefore prioritize the development of methods to better resolve such complexities, as exemplified by this atypical *C. sporogenes* strain from Vilauba.

In summary, through metagenomic screening and phylogenetic analyses, we detected DNA from *Clostridium* and *Mycolicibacterium* species in several Roman-era cattle remains. The discovery of a *Clostridium* strain closely related to known toxigenic lineages, along with its virulence-associated gene profile, suggests a potential for pathogenicity. However, whether this reflects part of the decaying necrobiome, postmortem environmental proliferation, or a genuine antemortem infection remains unresolved. Nevertheless, these findings challenge current assumptions in ancient pathogen research by drawing attention to a bacterium that blurs the lines between harmless commensal, environmental contaminant and potential pathogen. Rather than being dismissed as background noise, *C. sporogenes,* and other such bacterial taxa may represent a blind spot in our current understanding of livestock health. Future studies are required to address the complexity of these yet ambiguous taxa and tools which disentangle infection from infiltration will be essential for reconstructing a fuller picture of disease in the past.

## Supplementary Material

Supplementary_Tables.xlsx

Change_of_authorship_form.pdf

Supplementary_Figures.docx

Submitted_manuscript_with_changes_v4.docx

## Data Availability

Raw sequences generated in this study have been deposited in the European Nucleotide Archive under the accession: https://www.ebi.ac.uk/ena/browser/view/PRJEB85544 [108]. All supplementary figures and tables have been deposited in a recognized data repository (www.Figshare.com) under the accession: 10.6084/m9.figshare.28458068 [109].
