# Supplementary material for: Detection of Clostridium sporogenes in a Roman-era cattle mass grave at Vilauba
Source: Virulence. 2025 Oct 27;16(1):2580731. doi: 10.1080/21505594.2025.2580731 (PMC12587821; doi:10.1080/21505594.2025.2580731)
Supplement: Change_of_authorship_form.pdf [file KVIR_A_2580731_SM4763.pdf]

Change of authorship request form – Journals

Please complete this form fully; if it is not completed, it will not be reviewed and returned to you. Please note that if an authorship change is made for a journal that does not consider authorship changes, your submission may be automatically withdrawn and returned to you.

Where appropriate, the journal reserves the right to publish the authorship history of the article, including all previous author lists; by completing this form, authors grant permission for this information to be included in the published article.

If a request is made which is considered in breach of the journal's editorial policies, the journal reserves the right to inform the relevant authors' institution.

**Title of manuscript:**	Detection of *Clostridium sporogenes* in a Roman-era cattle mass grave at Vilauba
**Manuscript ID no.:**	256846944
**Journal Name:**	Virulence
**Total no. of authors added:**	1
**Total no. of authors removed:**	0

1. **Original author list at submission, in the order shown on the manuscript. Please indicate the corresponding author with a ***

Original author list at submission	First name(s)	Family name	Affiliation (institute/organisation, department)	Email address
1st author	Daniel Anton	Myburgh	Institute of Clinical Molecular Biology	d.myburgh@ikmb.uni-kiel.de
2nd author	Nicolas Antonio	da Silva	Institute of Clinical Molecular Biology	n.dasilva@ikmb.uni-kiel.de
3rd author	Magdalena	Haller-Caskie	Institute of Clinical Molecular Biology	magdalena.haller@gmail.com
4th author	Lídia	Colominas	Catalan Institute of Classical Archaeology	lcolominas@icac.cat
5th author	Pere	Castanyer	Grup de Recerca Arqueològica del Pla de l'Estany	pcastanyer@gencat.cat
6th author	Joan	Frigola	Museu Arqueològic de Banyoles, Banyoles	joanfrigola@hotmail.com
7th author	Joaquim	Tremoleda	Grup de Recerca Arqueològica del Pla de l'Estany	jtremoleda@gencat.cat
8th author	Daniel	Unterweger	Institute of Experimental Medicine, Kiel University and Max Planck Institute for Evolutionary Biology	d.unterweger@iem.uni-kiel.de

**9th author**	Almut	Nebel	Institute of Clinical Molecular Biology	a.nebel@mucosa.de
**10th author***	Ben*	Krause-Kyora*	Institute of Clinical Molecular Biology	b.krause-kyora@ikmb.uni-kiel.de

Please insert new rows if needed

2. Please provide an explanation for the change in authorship **(including any reasons for additions to, or removals from the original authorship list)**.

In response to a reviewer's comment, we consulted with Professor Christina Hölzel, a veterinarian, who provided valuable expertise in veterinary terminology and interpretation of our findings and thoroughly revised the manuscript. In recognition of these significant contributions, we have added Professor Hölzel as a co-author and updated the author contributions statement accordingly. No authors have been removed from the original authorship list.

3. **Proposed new authorship list on the current submission (including email addresses), in the order it should appear on the manuscript. Please indicate the corresponding author with an asterix (*).**

Proposed new author list and order	First name(s)	Family name	Affiliation (institution/organisation)	Email address	Please provide details of how this author contributed to the article & the reported findings.
**1st author**	Daniel Anton	Myburgh	Institute of Clinical Molecular Biology	d.myburgh@ikmb.uni-kiel.de	Generated and analysed the ancient DNA data. Interpreted the findings. Wrote the manuscript. Revised the manuscript.
**2nd author**	Nicolas Antonio	da Silva	Institute of Clinical Molecular Biology	n.dasilva@ikmb.uni-kiel.de	Generated and analysed the ancient DNA data. Interpreted the findings.
**3rd author**	Magdalena	Haller-Caskie	Institute of Clinical Molecular Biology	magdalena.haller@gmail.com	Generated and analysed the ancient DNA data.

**4th author**	Lídia	Colominas	Catalan Institute of Classical Archaeology	lcolominas@icac.cat	Conceptualization and design. Provided the skeletal material. Provided environmental, archaeological and historical context and interpretation. Revised the manuscript.
**5th author**	Pere	Castanyer	Grup de Recerca Arqueològica del Pla de l'Estany	pcastanyer@gencat.cat	Provided environmental, archaeological and historical context and interpretation.
**6th author**	Joan	Frigola	Museu Arqueològic de Banyoles, Banyoles	joanfrigola@hotmail.com	Provided environmental, archaeological and historical context and interpretation.
**7th author**	Joaquim	Tremoleda	Grup de Recerca Arqueològica del Pla de l'Estany	jtremoleda@gencat.cat	Provided environmental, archaeological and historical context and interpretation.
**8th author**	Christina	Hölzel	Institute for Animal Breeding and Husbandry	choelzel@tierzucht.uni-kiel.de	Contributed veterinary expertise. Revised the manuscript.
**9th author**	Daniel	Unterweger	Institute of Experimental Medicine, Kiel University and Max Planck Institute for Evolutionary Biology	d.unterweger@iem.uni-kiel.de	Interpreted the findings.

10th author	Almut	Nebel	Institute of Clinical Molecular Biology	a.nebel@mucosa.de	Interpreted the findings. Revised the manuscript.
11th author *	Ben*	Krause-Kyora*	Institute of Clinical Molecular Biology	b.krause-kyora@ikmb.uni-kiel.de	Conceptualization and design. Interpreted the findings. Revised the manuscript.

Please insert further rows if required

4. **All authors (unchanged, added and/or removed) must sign this declaration.**

Signatures can be in the form of Docusign, electronic certified signature, or handwritten signatures can be returned as an image file.

Typed names in the signature box **WILL NOT** be accepted unless accompanied by an additional email confirmation from that co-author agreeing to the changed author list and the explanation of changes outlined in Section 2. This email must come from the same email address assigned to that co-author in the above sections.

**Author name (first name, last name)**	**Declaration**	**I consent to being named in the acknowledgments if the authorship change is rejected**	**Signature (if typed please ensure confirmation via email is attached)**	**Date signed**
Daniel Anton Myburgh	**I agree to the new authorship list and contributions shown above in section 3, for the reasons outlined in section 2.**	Not applicable	[illegible]	01. 10. 2025
Nicolas Antonio da Silva	**I agree to the new authorship list and contributions shown above in section 3, for the reasons outlined in section 2.**	Not applicable	Nicolas A. Silva	01.10.2025
Magdalena Haller-Caskie	**I agree to the new authorship list and contributions shown above in section 3, for the reasons outlined in section 2.**	Not applicable	Magdalena Haller-Caskie	01.10.2025

Lídia Colominas	I agree to the new authorship list and contributions shown above in section 3, for the reasons outlined in section 2.	Not applicable	[illegible]	02.10 2025
Pere Castanyer	I agree to the new authorship list and contributions shown above in section 3, for the reasons outlined in section 2.	Not applicable	[illegible]	02.10.2025
Joan Frigola	I agree to the new authorship list and contributions shown above in section 3, for the reasons outlined in section 2.	Not applicable	[illegible]	02-10-2025
Joaquim Tremoleda	I agree to the new authorship list and contributions shown above in section 3, for the reasons outlined in section 2.	Not applicable	[illegible]	02.10.2025
Christina Hölzel	I agree to the new authorship list and contributions shown above in section 3, for the reasons outlined in section 2.	[illegible]	[illegible]	6.10.25
Daniel Unterweger	I agree to the new authorship list and contributions shown above in section 3, for the reasons outlined in section 2.	Not applicable	[illegible]	02.10.2025
Almut Nebel	I agree to the new authorship list and contributions shown above in section 3, for the reasons outlined in section 2.	Not applicable	Almut Nebel	01.10.2025
Ben Krause-Kyora*	I agree to the new authorship list	Not applicable	[illegible]	01.10.2025

	**and contributions shown above in section 3, for the reasons outlined in section 2.**			

For submissions to journals using the CRediT role taxonomy, please also complete the next page. If the journal uses the CRediT role taxonomy, this will be clearly stated on the journal homepage.

5. **CRediT roles (if applicable) for proposed author list (please tick the appropriate roles).** For more information on Credit, please consult: https://credit.niso.org/contributor-roles-defined/.

		Conceptualization	Data Curation	Formal analysis	Funding acquisition	Investigation	Methodology	Project administration	Resources	Software	Supervision	Validation	Visualization	Writing- original draft	Writing- review & editing
Author names	Daniel Anton Myburgh			x		x							x	x	x
	Nicolas Antonio da Silva			x		x							x		x
	Magdalena Haller-Caskie			x		x									
	Lídia Colominas	x							x						x
	Pere Castanyer								x						x
	Joan Frigola								x						x
	Joaquim Tremoleda								x						x
	Christina Hölzel					x									x
	Daniel Unterweger					x									x
	Almut Nebel				x	x		x			x				x

	Ben Krause-Kyora*	x			x	x		x	x		x				x
