# Supplementary material for: Detection of Clostridium sporogenes in a Roman-era cattle mass grave at Vilauba
Source: Virulence. 2025 Oct 27;16(1):2580731. doi: 10.1080/21505594.2025.2580731 (PMC12587821; doi:10.1080/21505594.2025.2580731)
Supplement: Supplementary_Figures.docx [file KVIR_A_2580731_SM4762.docx]

Supplementary Figures for:

**Detection of *Clostridium sporogenes* in a Roman-era cattle mass grave at Vilauba**

Daniel Anton Myburgh, Nicolas Antonio da Silva, Magdalena Haller-Caskie, Lídia Colominas, Pere Castanyer, Joan Frigola, Joaquim Tremoleda, Christina Hölzel, Daniel Unterweger, Almut Nebel, Ben Krause-Kyora*

*Corresponding author email: b.krause-kyora@ikmb.uni-kiel.de

**This PDF file includes:**

Supplementary Figure S1. Genetic sex determination of cattle samples

Supplementary Figure S2. Phylogenetic analysis of cattle mitochondrial haplogroups

Supplementary Figure S3. Deamination plots generated from the mapping of bacterial species detected in metagenomic pathogen screening

Supplementary Figure S4. Percentage coverage of clostridial virulence factors

Supplementary Figure S5. Deamination plots of mapping to “*Mycobacterium gallinarum”* JCM 6399 and *Mycolicibacterium gadium* JCM 12688

**Other supplementary materials for this manuscript include:**

Supplementary Tables S1 – S13


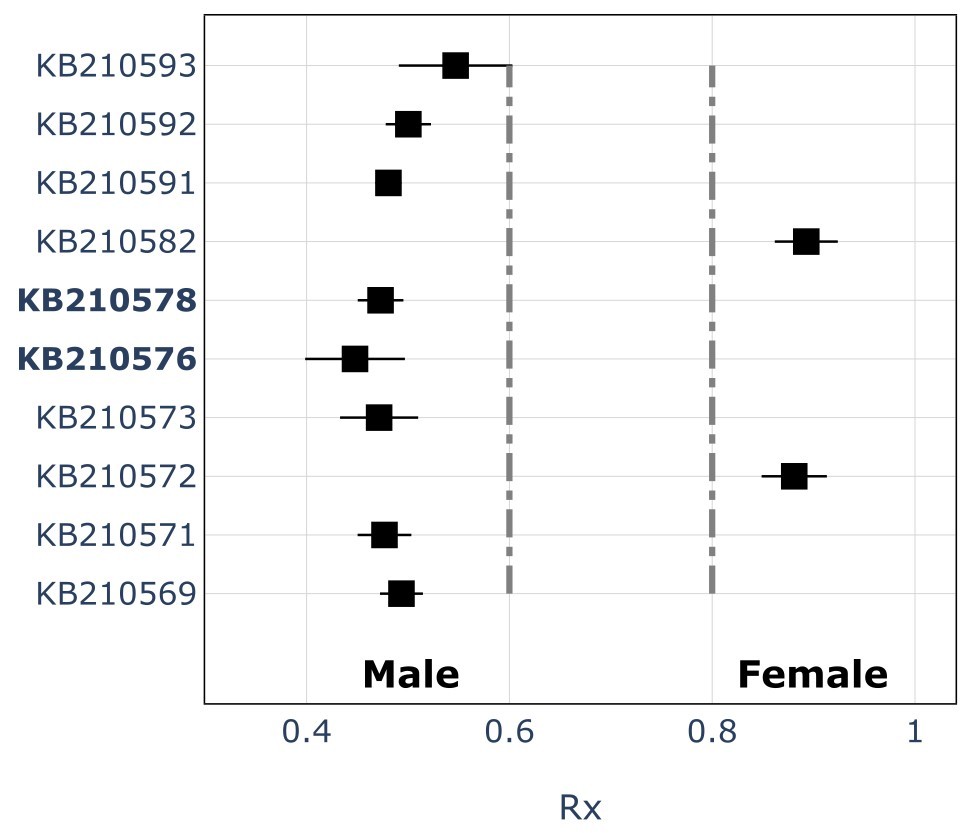


**Supplementary Figure S1. Genetic sex determination of cattle samples**

The x-axis displays the ratio of reads aligned to the X chromosome compared to the ratio of reads aligned to all autosomes (Rx). Dashed lines along the y-axis represent the thresholds used for sex assignment. The error bars represent the 95% confidence interval. Bold sample IDs indicate samples with osteometric data for sex determination.

**
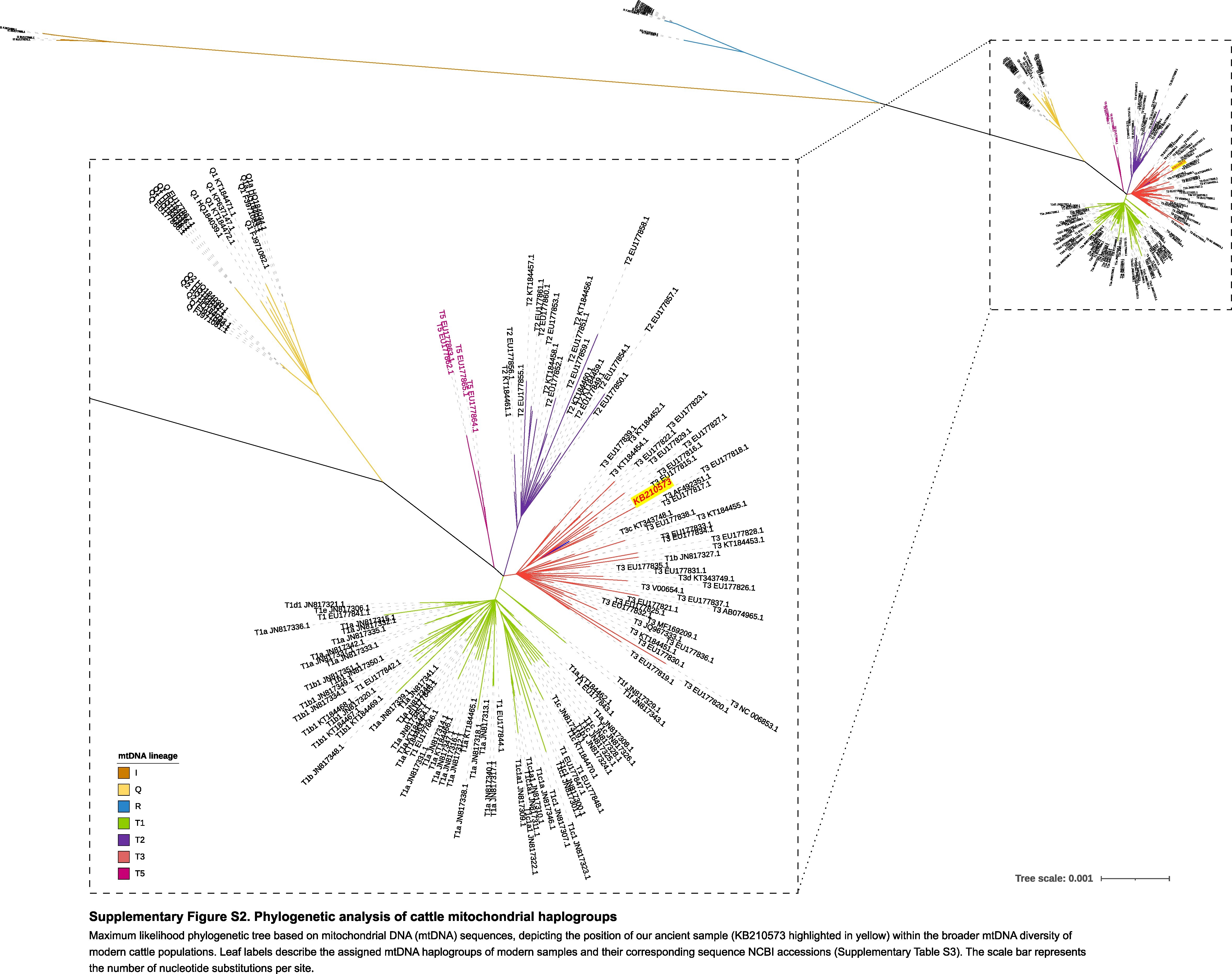
**

**S3a**


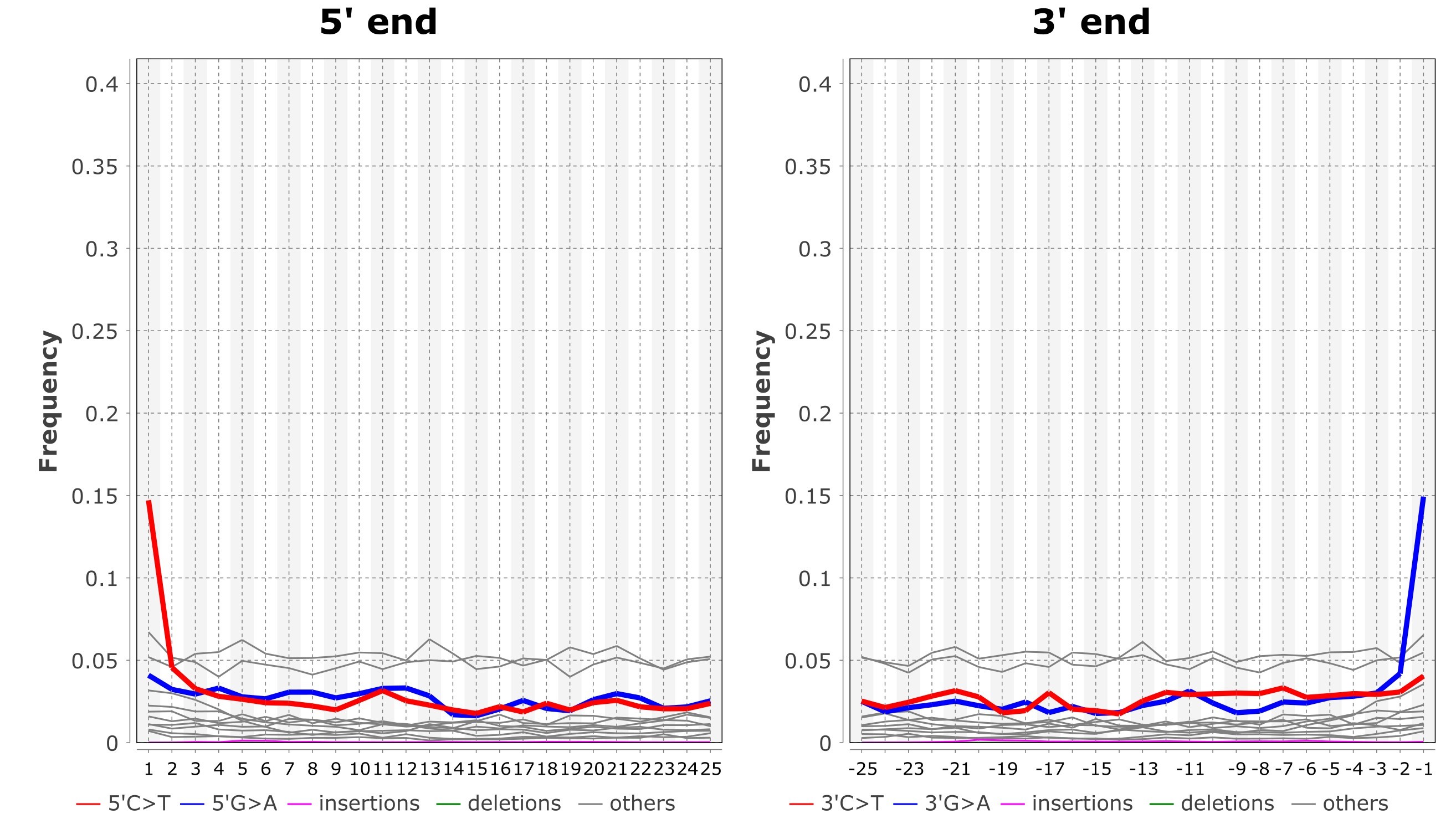


**S3b**


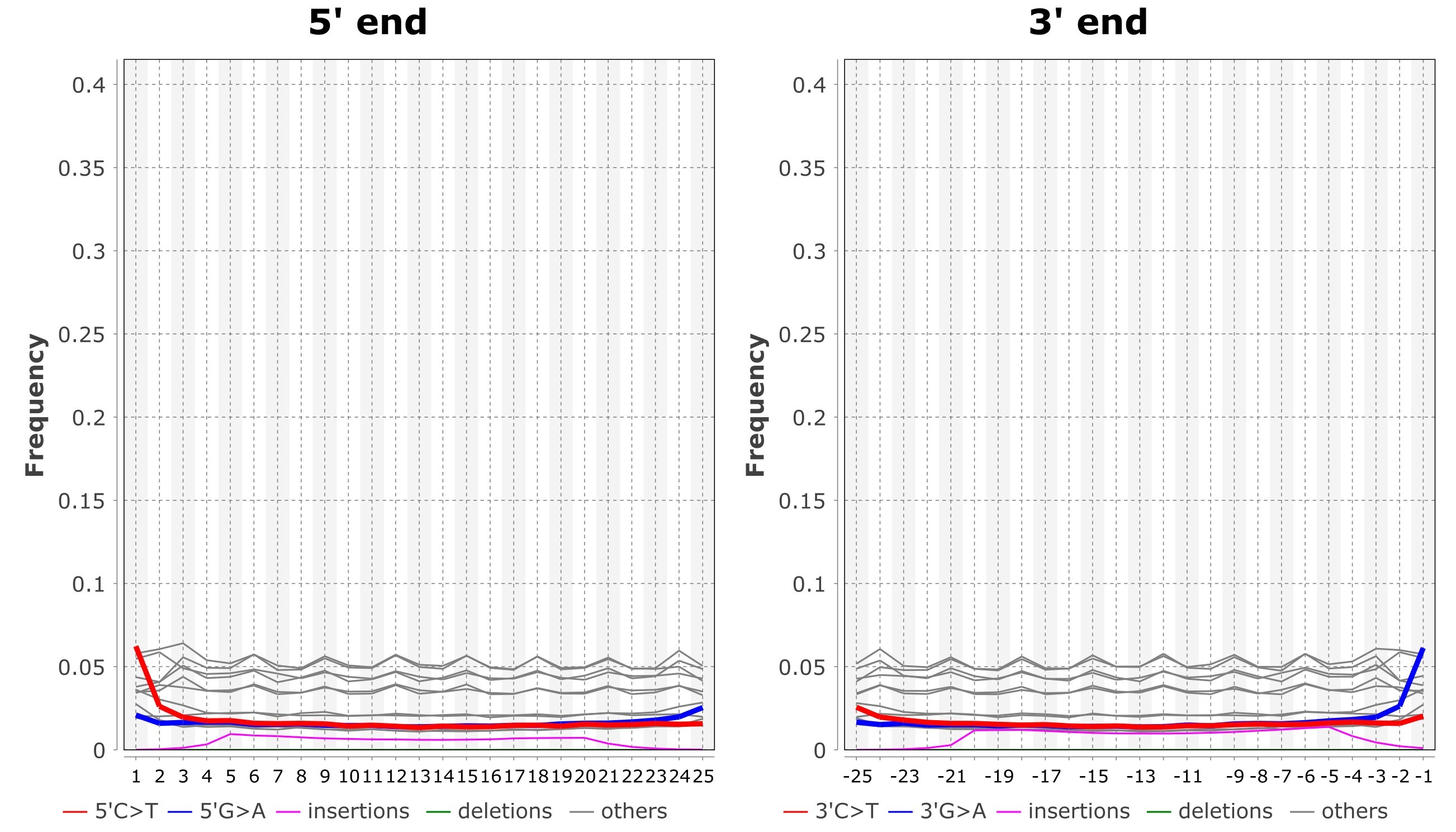


**S3c**


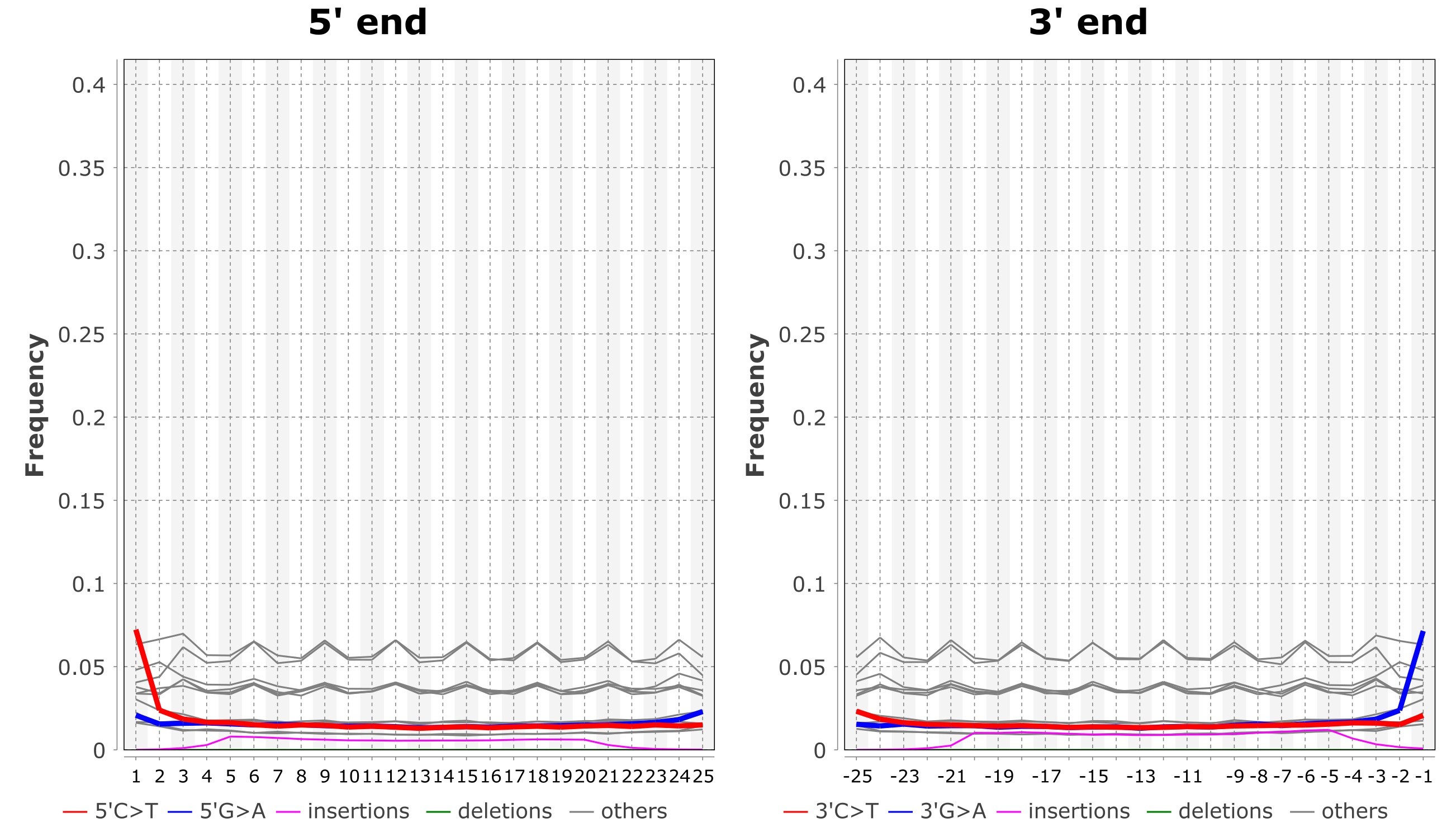


**S3d**


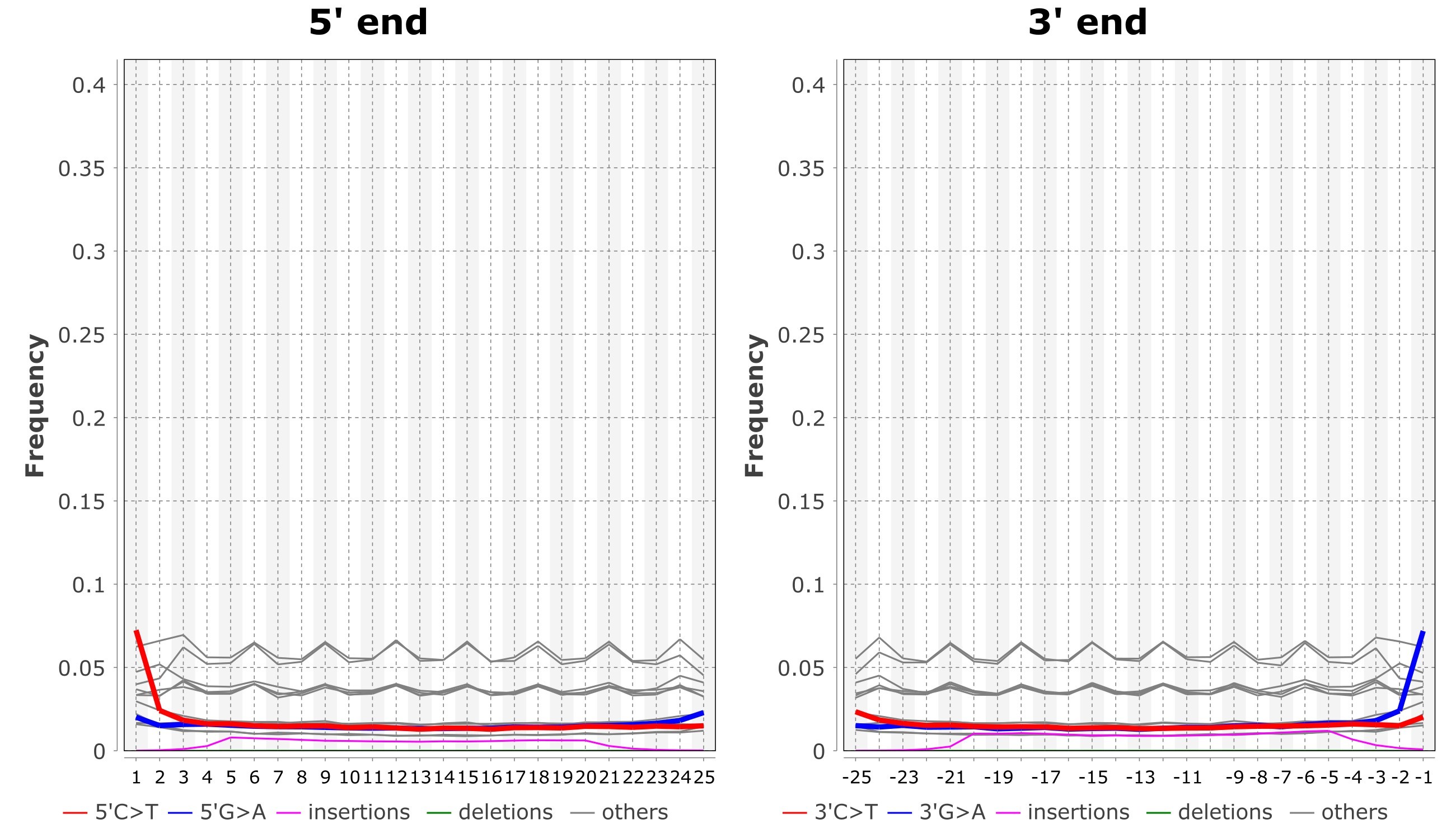


## Supplementary Figure S3. Deamination plots generated from the mapping of bacterial species detected in metagenomic pathogen screening

DNA deamination plots generated using DamageProfiler v.1.1 [1]. All plots were generated from mapping data using reads from deeper sequenced sample KB210573. **S3a.** Mapped to *Clostridium sporogenes* AM1195 (NCBI RefSeq: NZ_CP013701.1). **S3b.** Mapped to *Mycobacterium avium subsp. paratuberculosis* K-10 (NCBI RefSeq: NZ_CP106873.1). **S3c.** Mapped to “*Mycobacterium gallinarum”* JCM 6399 (NCBI RefSeq: NZ_AP022601.1). **S3d.** Mapped to *Mycolicibacterium gadium* JCM 12688 (NCBI RefSeq: NZ_AP022608.1).


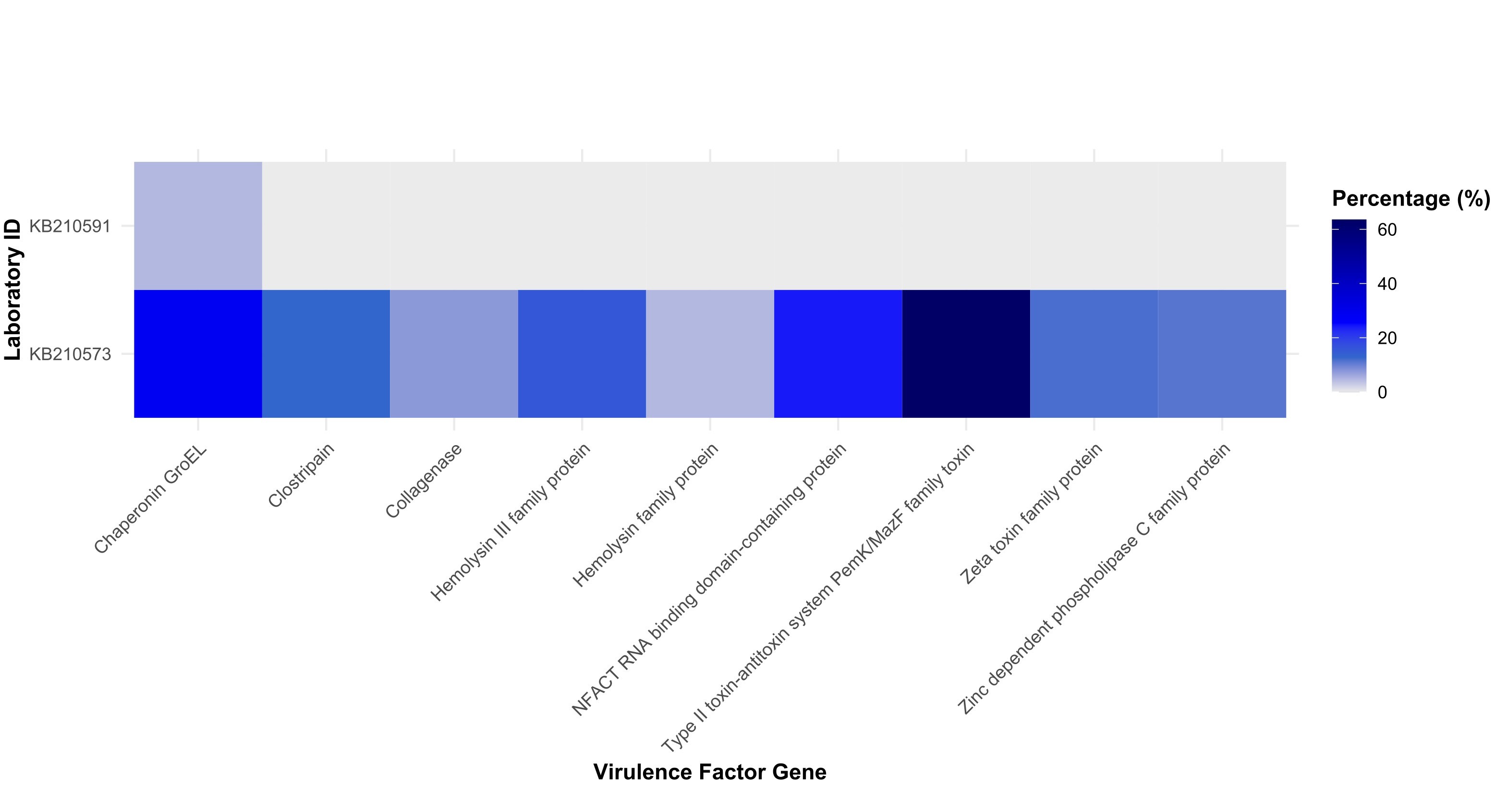


## Supplementary Figure S4. Percentage coverage of clostridial virulence factors

Heatmap generated using the R package ggplot2 [2] indicating the percentage of coverage of clostridial virulence genes. Legend indicates the percentage of coverage and the corresponding shade of colour. Only samples which had coverage of at least one clostridial virulence gene are displayed. Only clostridial virulence genes with coverage in at least one sample are displayed. Only the highest percentage coverage is displayed for each of the tested virulence genes from the different *Clostridium* species.

**S5a**


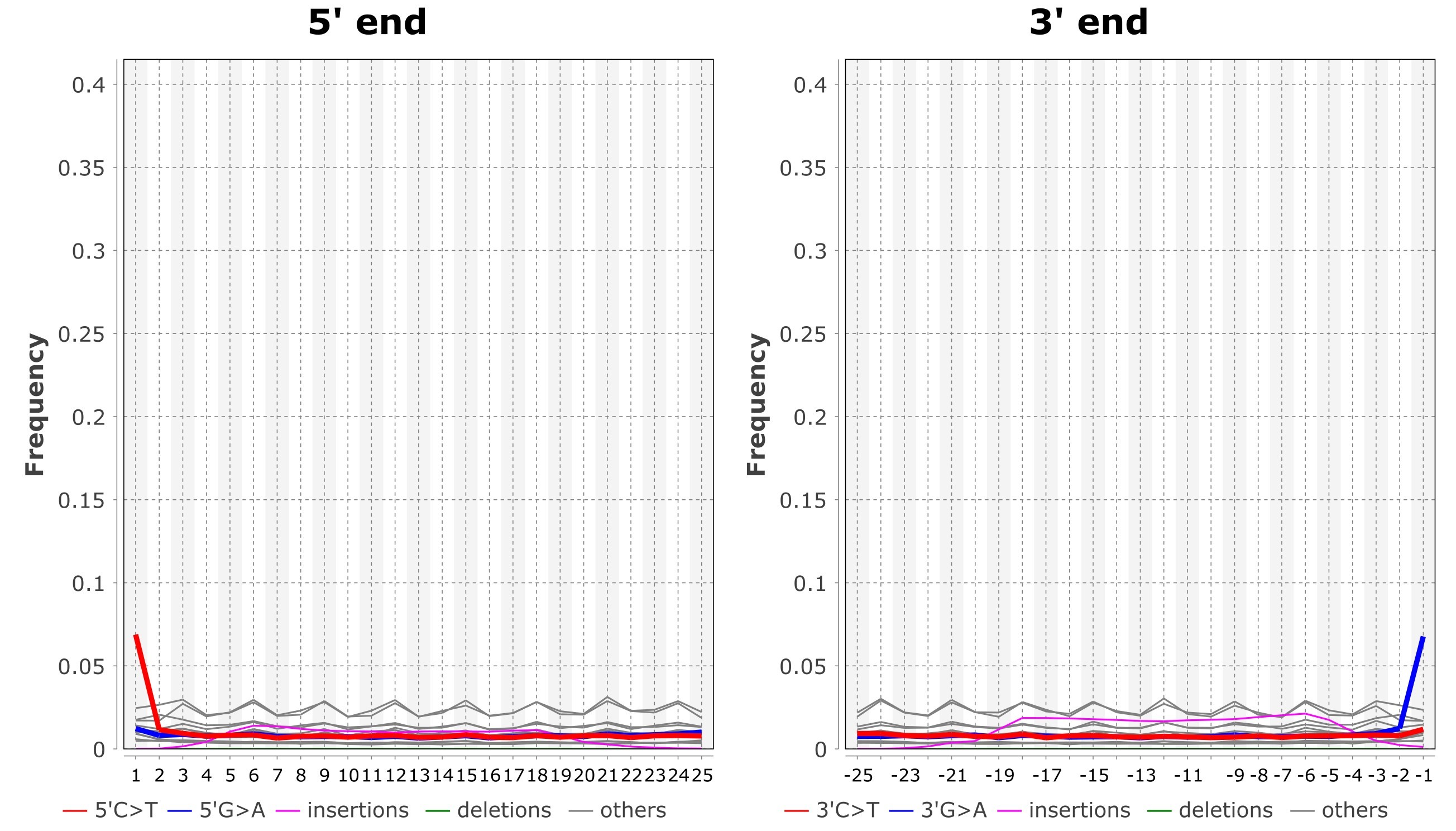


**S5b**


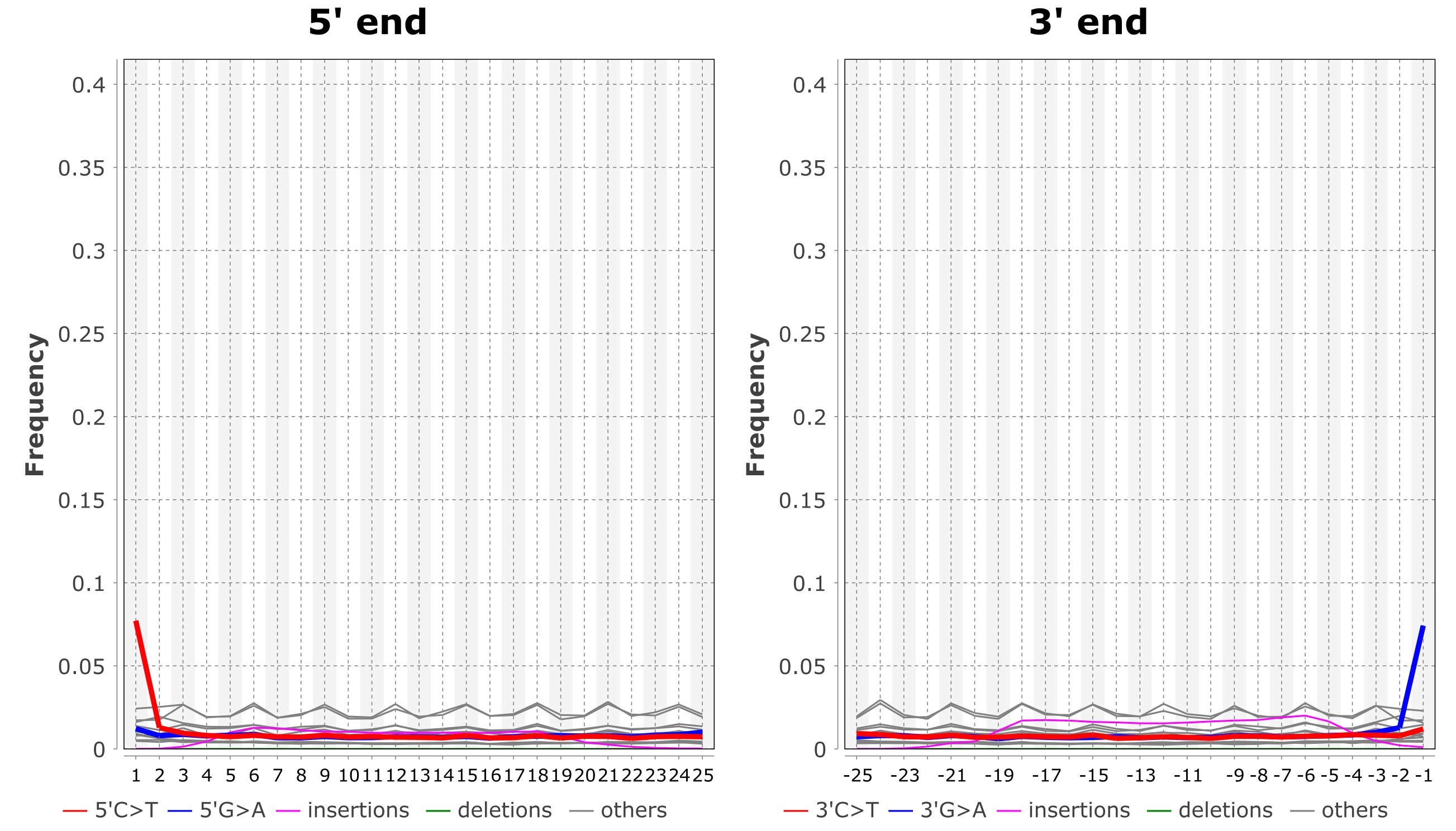


## Supplementary Figure S5. Deamination plots of mapping to “*Mycobacterium gallinarum”* JCM 6399 and *Mycolicibacterium gadium* JCM 12688

DNA deamination plots generated using DamageProfiler v.1.1 [1]. All plots were generated from filtered mapping data using reads from deeper sequenced sample KB210573. **S5a.** Mapped to “*Mycobacterium gallinarum”* JCM 6399 (NCBI RefSeq: NZ_AP022601.1). **S5b.** Mapped to *Mycolicibacterium gadium* JCM 12688 (NCBI RefSeq: NZ_AP022608.1).

**References**

[1] Neukamm J, Peltzer A, Nieselt K. DamageProfiler: fast damage pattern calculation for ancient DNA. Bioinformatics. 2021;37(20):3652–3653.

[2] Wilkinson L. ggplot2: Elegant Graphics for Data Analysis by WICKHAM, H. Biometrics. 2011;67(2):678–679.
